# Interfacial Heterojunction Enables High Efficient PbS Quantum Dot Solar Cells

**DOI:** 10.1002/advs.202402756

**Published:** 2024-05-02

**Authors:** Li Zhang, Yong Chen, Shuang Cao, Defei Yuan, Xu Tang, Dengke Wang, Yajun Gao, Junjie Zhang, Yongbiao Zhao, Xichuan Yang, Zhenghong Lu, Quli Fan, Bin Sun

**Affiliations:** ^1^ State Key Laboratory of Organic Electronics and Information Displays & Institute of Advanced Materials (IAM) School of Material Science and Engineering Nanjing University of Posts and Telecommunications (NJUPT) 9 Wenyuan Rd. Nanjing 210023 China; ^2^ Department of Physics Center for Optoelectronics Engineering Research Yunnan University Kunming 650091 China; ^3^ LONGi Central R&D Institute LONGi Green Energy Technology Co. Xi'an China; ^4^ Institute of Artificial Photosynthesis State Key Laboratory of Fine Chemicals DUT−KTH Joint Education and Research Centre on Molecular Devices Dalian University of Technology (DUT) 2 Linggong Rd. Dalian 116024 China

**Keywords:** charge extraction, colloidal quantum dots, interface heterojunction, solar cells

## Abstract

Colloidal quantum dots (CQDs) are promising optoelectronic materials for solution‐processed thin film optoelectronic devices. However, the large surface area with abundant surface defects of CQDs and trap‐assisted non‐radiative recombination losses at the interface between CQDs and charge‐transport layer limit their optoelectronic performance. To address this issue, an interface heterojunction strategy is proposed to protect the CQDs interface by incorporating a thin layer of polyethyleneimine (PEIE) to suppress trap‐assisted non‐radiative recombination losses. This thin layer not only acts as a protective barrier but also modulates carrier recombination and extraction dynamics by forming heterojunctions at the buried interface between CQDs and charge‐transport layer, thereby enhancing the interface charge extraction efficiency. This enhancement is demonstrated by the shortened lifetime of carrier extraction from 0.72 to 0.46 ps. As a result, the resultant PbS CQD solar cells achieve a power‐conversion‐efficiency (PCE) of 13.4% compared to 12.2% without the heterojunction.

## Graphical abstract

Interfacial heterojunction not only acts as a protective barrier but also modulates carrier recombination, thereby enhancing the interface charge extraction efficiency, achieving PCE of 13.4%.

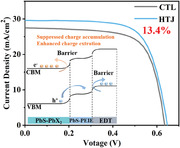

## 1 Introduction

Colloidal quantum dots (CQDs) are promising optoelectronic materials due to their tunable bandgap and solution processing compatibility, making them highly attractive for solar cell applications.^[^ ^1^, ^2^, ^3^, ^4^, ^5^, ^6^ ^]^ The recent achievements of surface passivation, interface modulation, and device architecture have led to improved CQD solar cell performance, and enabled power conversion efficiencies (PCE) above 13% for lead sulfide (PbS) CQDs.^[^ ^2^, ^7^, ^8^, ^9^, ^10^, ^11^, ^12^, ^13^, ^14^ ^]^

The conventional architecture of CQD solar cells typically comprises a transparent cathode, an electron transport layer (ETL), a light‐absorbing active layer, a hole transport layer (HTL), and a metal anode. In many high‐performing CQD solar cells, 1,2‐ethanedithiol (EDT) is utilized as the ligand in the CQD HTL, employing a solid film ligand‐exchange method.^[^ ^7^, ^8^, ^15^, ^16^, ^17^, ^18^, ^19^, ^20^ ^]^ However, the high reactivity of EDT during ligand exchange can introduce significant surface defects, which act as charge recombination centers and diminish the efficiency of charge extraction at the interface between the HTL and active layer.^[^ ^16^, ^17^, ^21^, ^22^, ^23^, ^24^ ^]^

Currently, the formation of the incorporation of carrier concentration gradient layers is a proven strategy to enhance the separation efficiency of electrons and holes, as well as the efficiency of carrier extraction.^[^ ^13^, ^25^, ^26^, ^27^, ^28^, ^29^, ^30^, ^31^, ^32^ ^]^ Nevertheless, the presence of EDT at the back‐junction still poses a limitation on charge extraction.^[^ ^33^, ^34^, ^35^ ^]^ Although attempts have been made to replace EDT‐passivated CQD HTLs with carboxylic acids and organic p‐type semiconductors, the highest‐performing devices continue to utilize EDT‐passivated CQD‐based HTLs.^[^ ^22^, ^36^, ^37^, ^38^, ^39^, ^40^, ^41^, ^42^, ^43^, ^44^, ^45^, ^46^ ^]^

**Figure 1:**
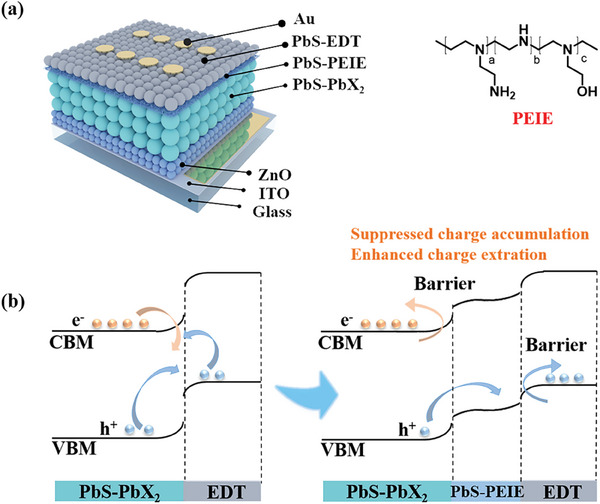
Device structure and energy level arrangement. a) Schematic diagram of PEIE modulated PbS‐CQD solar cell devices, PEIE molecule structure. b) Schematic diagram of PbS‐PEIE interface heterojunction layer introduction in devices.

Here, we propose the strategy of interfacial heterojunction modulation to improve charge extraction and suppress the non‐radiative recombination losses at the interface between CQDs and charge‐transport layer. By incorporating a thin layer of polyethyleneimine (PEIE), we aim to safeguard the CQD interface. This protective barrier not only shields the interface but also alters the dynamics of carrier recombination and extraction by fostering heterojunctions formation at the buried interface between CQDs and charge‐transport layer, thereby enhancing the interface charge extraction efficiency (**Figure** **1**; Figure S1, Supporting Information). The formation of interfacial heterojunction modulation is demonstrated by the shortened carrier extraction lifetime, as confirmed by the observation of trap‐to‐ground‐state recombination using time‐resolved spectroscopy. Consequently, this approach translates into CQD solar cells exhibiting higher power conversion efficiencies (PCEs) of 13.4% compared to 12.2% without the heterojunction. Overall, our findings not only unveil an efficient method for enhancing charge extraction efficiency but also pave the way for further improving interface quality through the mechanism of interfacial heterojunction modulation.

## 2 Results and Discussion

**Figure 2:**
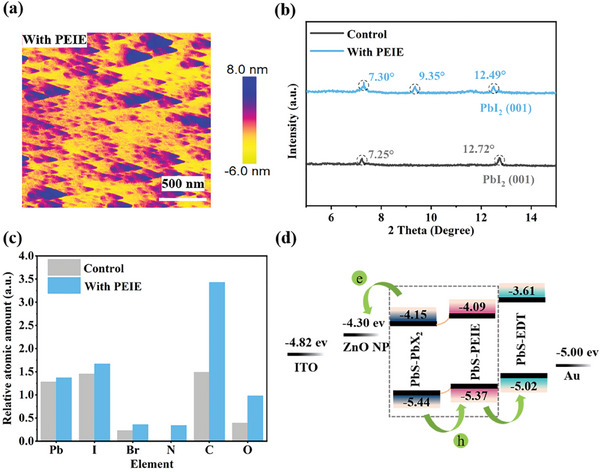
Characterization of the contact interface after absorption layer modulated by PEIE. a) AFM images of PEIE (PbS‐PbX_2_+PEIE) films on ITO substrates. b) XRD spectra of control (PbS‐PbX_2_) and with PEIE (PbS‐PbX_2_+PEIE) films. c) Elemental ratios of PbS‐PbX_2_ CQDs with and without PEIE modulation from X‐ray photoelectron spectroscopy (XPS). d) Energy level alignment of the functional layers.

We prepared PbS‐CQD active layers by spin coating a ligand‐exchanged lead halide‐passivated CQDs (PbS‐PbX_2_ CQDs) in a butylamine solution.^[^ ^14^ ^]^ Subsequently, we spin‐coated the PEIE solution in isopropanol onto the PbS‐PbX_2_ CQD films, serving as a protective layer for the CQD surface (Figure S1, Supporting Information). Initially, we characterized the film morphology utilizing atomic force microscopy (AFM). Upon spin‐coating the PEIE, distinctive triangular star‐shaped structures emerged on the PbS‐PbX_2_ surface (**Figure** **2**a). The control film was not observed (Figure S2a, Supporting Information). This observation suggests that the PEIE has effectively coated the CQD surface, potentially forming new structures. Subsequent X‐ray diffraction (XRD) measurements were conducted to analyze the newly formed components. A characteristic peak at 9.35° was identified (Figure 2b), which we attributed to the presence of 2D perovskites formed by the ligands PbI_2_ on the CQD surface and PEIE.^[^ ^47^, ^48^ ^]^

To validate this assumption, we prepared films with PbI_2_+PEIE, PbBr_2_+PEIE, and PbI_2_+PbBr_2_+PEIE, and subjected them to XRD analysis (Figure S3, Supporting Information). The similar 2D peak observed in all films indicated that the triangular structures are indeed 2D perovskites formed by the interaction of PEIE with PbX_2_ (where X = Br^−^, I^−^). We also checked the absorption spectra of PbI_2_ and PbI_2_+PEIE films (Figure S4, Supporting Information), absorption spectra analysis of PbI_2_ and PbI_2_+PEIE films revealed a redshift in the cut‐off edge absorption from 532 to 548 nm, consistent with the formation of 2D perovskites.^[^ ^49^ ^]^ Furthermore, we conducted AFM analysis of the film morphology after cleaning it with acetonitrile (ACN), revealing identical star‐shaped structures (Figure S2b, Supporting Information) on the PbS‐PbX_2_+PEIE film compared to the film without ACN treatment. We attribute this observation to the relative stability of the 2D perovskite in ACN,^[^ ^50^, ^51^ ^]^ consistent with the XRD findings.

To gain a comprehensive understanding of how PEIE protects the CQD film surface, we conducted X‐ray photoelectron spectroscopy (XPS) analyses on the CQDs. Elemental ratios extracted from XPS data (Figure 2c; Figure S5, Supporting Information), normalized to the S 2p peak, showed a significant increase in N/S, C/S, and O/S ratios on the CQD surface after the modulation, supporting the presence of a PEIE coating. Additionally, a slight increase in Pb/S and I/S ratios was noted, agreeing with the formation of 2D perovskites on the film surface (Figure 2c).

We also investigated whether the formation of 2D perovskites affects the optoelectronic properties of CQD solids. We observed a broader absorption in the films after PEIE modulation (Figure S6, Supporting Information). This broadening could be attributed to the unchanged primary CQD solids and the CQD surface experiencing a reduced inter‐dot distance due to the formation of perovskite on the surface. This perovskite process was previously identified in Sargent's work, where acetonitrile influences the entire film, while IPA solvent specifically affects the CQD film surface.^[^ ^2^, ^47^, ^48^, ^52^ ^]^ Subsequently, we conducted UV photoelectron spectroscopy (UPS) measurements to examine the band alignment of CQD films with and without PEIE modulation. The Fermi energy (*E_F_*) levels for control and PEIE films were calculated to be 4.61 and 4.47 eV, respectively. Additionally, their valence band maximum (VBM) levels were −5.44 and −5.37 eV (Figure S7, Supporting Information), respectively, indicating the potential formation of a type II heterojunction that could facilitate charge separation (Figure 2d).

**Figure 3:**
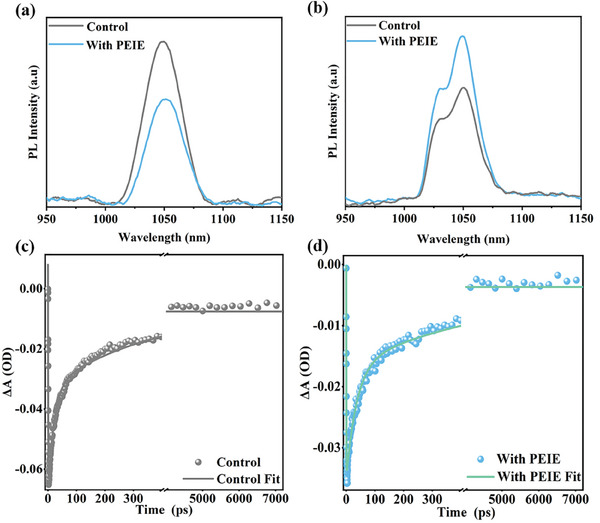
Charge carrier extraction and recombination. a,b) Steady‐state photoluminescence spectra (PL) of control (PbS‐PbX_2_) and with PEIE (PbS‐PbX_2_+PEIE) films and corresponding device, respectively. c,d) The TA spectra kinetics of control (PbS‐PbX_2_) and PEIE (PbS‐PbX_2_+PEIE) film samples. The detection wavelength is at the maximum absorption of the bleaching peak. (Excitation wavelength 500 nm, excitation intensity 180 µw.)

To further substantiate the claim of improved carrier extraction efficiency, we conducted photoluminescence (PL) measurements. The PL intensity of the film decreased after PEIE treatment (**Figure** **3**a), which we attribute to the formation of a type II heterojunction or damage to the passivation of the CQD surface. To rule out the possibility of a surface passivation issue, we performed PL measurements on CQD films within diode devices, including electron transport layer (ETL)‐ZnO, hole transport layer (HTL)‐PbS‐EDT, and electrodes‐Au. Interestingly, we observed an increased PL intensity with PEIE treatment (Figure 3b), indicating enhanced surface passivation and reduced trap density.^[^ ^53^, ^54^, ^55^ ^]^ Combining the band alignments obtained from UPS and the observed changes in PL intensity, we conclude that the PEIE interface modulation indeed forms a type II interfacial heterojunction.

To further confirm whether the interfacial heterojunction facilitates charge separation, we conducted ultrafast transient absorption (TA) measurements to monitor the kinetics of charge‐carrier extraction. Figure S8 (Supporting Information) presents the contour plots and transient absorptions of the femtosecond TA (fs‐TA) spectra for control (PbS‐PbX_2_) and with PEIE (PbS‐PbX_2_+PEIE) samples, showing a negative photobleaching signal around ≈980 nm in the band edge of PbS. This signal is attributed to band filling by photoexcited carriers in PbS.^[^ ^8^, ^56^, ^57^, ^58^ ^]^ The femtosecond TA kinetics of control and PEIE samples were plotted (Figure 3c,d), and the curves were fitted using a triexponential function (Table S1, Supporting Information). The carrier extraction lifetimes (*τ_1_*) for control and PEIE samples were found to be 0.72 and 0.46 ps, respectively, indicating enhanced extraction/injection of hot carriers at the interface modulated by PEIE.^[^ ^58^, ^59^ ^]^ The trap capture lifetime (*τ_2_*) for control and with PEIE samples was found to be 20.28 and 36.65 ps, respectively, while the nonradiative recombination lifetime (*τ_3_*) was found to be 352.53 and 559.64 ps, respectively.^[^ ^7^, ^45^, ^58^ ^]^ These results suggest a significant reduction in defects and nonradiative recombination after the introduction of PEIE, consistent with the findings mentioned earlier.

**Figure 4:**
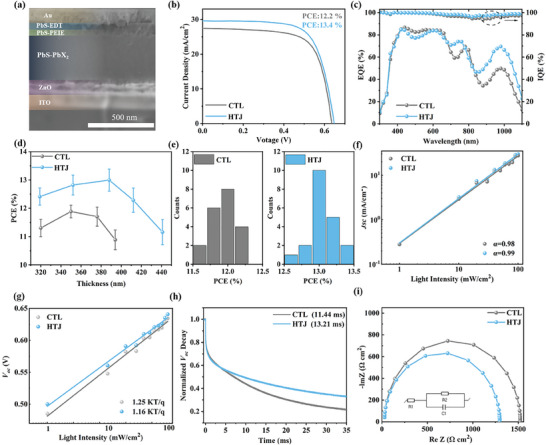
Photovoltaic performance, charge carrier transport and recombination of PbS CQD solar cells. a) Cross‐sectional SEM image of the HTJ (heterojunction PbS‐PbX_2_+PEIE CQD solar cell) devices. b) Current—voltage (*J*–*V*) curves of the best CTL (control PbS‐PbX_2_ CQD solar cell) and HTJ devices. c) External quantum efficiency (EQE) curves and internal quantum efficiency (IQE) curves of optimal CTL and HTJ devices. d) The relationship between PCE and CQDs thickness e) PCE statistical diagrams based on 20 devices. f,g) *J_sc_* and *V_oc_* versus light intensity of CTL and HTJ devices, respectively. h) TPV curves of CTL and HTJ devices. i) Impedance spectroscopy analysis of CTL and HTJ devices under illumination.

In pursuit of leveraging the improved charge extraction, we fabricated solar cells (**Figure** **4**a). The best‐performing HTJ (heterojunction PbS‐PbX_2_+PEIE CQD solar cell) devices were achieved with an active layer thickness of 390 nm (Figure 4a displays a PbS‐PbX_2_ film of ≈340, ≈50 nm of PbS‐PEIE interface layers, and ≈50 nm of EDT‐exchanged PbS CQD films as a hole transport layer), CTL (control PbS‐PbX_2_ CQD solar cell) devices optimal thickness of ≈350 nm (Figure S9, Supporting Information). This is consistent with the investigation of the thickness‐dependent PCE for the without and with PEIE CQD solar cells (Figure 4d). These HTJ devices exhibited a champion power conversion efficiency (PCE) of 13.4%, with open‐circuit voltage (*V_oc_*), current density (*J_sc_*), and fill factor (*FF*) values of 0.65 V, 29.6 mA·cm^−2^, and 69.7%, respectively. The best CTL devices achieved a PCE of 12.2%, with *V_oc_*, *J_sc_*, and FF values of 0.64 V, 27.5 mA·cm^−2^, and 69.5%, respectively (Figure 4b; Table S2, Supporting Information). The *J_sc_* values obtained from the *J*–*V* curves align with the integrated *J_sc_* values from the external quantum efficiency (EQE) measurements (Figure 4c; Figure S10, Supporting Information): the integrated *J_sc_* values for HTJ and CTL devices are 29.5 and 27.4 mA cm^−2^, respectively. The internal quantum efficiency (IQE), obtained by dividing the EQE at 0 V by the EQE at −1 V (Figure S10, Supporting Information),^[^ ^60^, ^61^ ^]^ indicates that HTJ devices have an IQE near unity across the entire spectral range, while CTL devices exhibit lower IQE values in the range of 700–1100 nm (Figure 4c). The interfacial heterojunction strategy enhances PCE due to improved charge collection at the back junction, aligning with the improved IQE in the long wavelength range where short‐wavelength light is predominantly absorbed at the front and long‐wavelength light is primarily absorbed at the back of the devices.^[^ ^2^, ^60^, ^61^ ^]^ Meanwhile, HTJ device display a desired reproducibility (Figure 4e). Furthermore, we measure the stabilized power output stability of unencapsulated device at a maximum power point under a continuous AM 1.5 G illumination in a N_2_ condition. (Figure S11a, Supporting Information). After 200 h aging, the HTJ devices retained up 90% of its initial PCE, whereas the reference dropped to 80% only after 100 h. In addition, devices can keep working with ≈90% of its initial PCE after 1000 h aging in glovebox conditions, and HTJ device is also better than the CTL device (Figure S11b, Supporting Information).

We investigated the charge carrier transport and recombination processes of the devices by measuring the light intensity‐dependent *J_sc_* and *V_oc_*. The ideality factors, derived from the slope of the aforementioned semi‐log plots of intensity‐dependent *J_sc_* (Figure 4f,g), are 0.98 for the control group and 0.99 for PEIE. Additionally, the ideality factors extracted from the light intensity‐dependent *V_oc_* are 1.25 for the control group and 1.16 for HTJ devices, respectively, which are close to 1. This suggests that the extraction of charge carriers is primarily limited by bimolecular recombination. The ideality factors for HTJ devices are even closer to 1, indicating that these devices experience less trap‐assisted recombination. This finding is consistent with the improved surface passivation and enhanced charge extraction capability demonstrated earlier.^[^ ^27^, ^62^ ^]^

To validate the reduced trap density in HTJ devices, we performed transient photovoltage (TPV) measurements under open‐circuit conditions with 1 sun light bias (Figure 4h). The longer lifetimes indicated fewer trap states.^[^ ^27^, ^62^ ^]^ The HTJ devices exhibited a slower *V_oc_* decay time (13.21 ms) compared to the CTL devices (11.44 ms), indicating fewer trap states in the HTJ devices, which aligns with the aforementioned results. Subsequently, electrochemical impedance spectroscopy (EIS) was employed to study the charge‐transfer resistance (*R_tr_*) and series resistance (*R_s_*) in the PbS CQDs devices (Figure 4i; Table S3, Supporting Information). Apparently, the *R_s_* of HTJ device exhibits a lower value of 28.04 Ω compared to that of control device (28.65 Ω). Meantime, the HTJ device also shows a *R_tr_* (1268 Ω) that is lower than that of the control device (1493 Ω), which indicates more efficient interfacial charge transfer to higher PCE.^[^ ^27^, ^43^ ^]^

## 3 Conclusion

In summary, we have presented a new approach for enhancing carrier transport and reducing interface recombination in CQD devices through interfacial heterojunction modulation. This modulation, achieved via PEIE, creates a 2D passivation structure at the interface. These improvements lead to increased charge extraction and transport efficiency, passivation of partial defects, and suppression of non‐radiative charge recombination. The HTJ CQD devices exhibit higher photocurrent (*J_sc_*) and fill factor (*FF*) due to the improved separation and transport of photoexcited carriers along distinct physical paths. Consequently, the resulting solar cells achieve the improved PCE in CQDs photovoltaic devices (13.4%). This interfacial heterojunction modulation strategy not only enhances CQDs quality but also improves device charge transfer, providing a promising pathway for developing high‐performance CQD solar cells.

## 4 Experimental Section

### PbS‐PbX_2_ Ligand Exchange

PbI_2_ (0.1 m), PbBr_2_ (0.04 m), and ammonium acetate (0.04 m) were dissolved in 20 mL DMF for a standard ligand exchange. The 7 mg mL^−1^ PbS (20 mL) solution was mixed with the DMF solution. Shake vigorously for 2 min. During this period, the ligand exchange event causes the upper liquid to turn colorless and the lower liquid to turn black. Rinse five times with 20 mL of *n*‐hexane. After adding toluene at a volume ratio of 1:3, the precipitate was finally achieved, and the PbS quantum dots were dried in a vacuum for 20 min. After that, the dried quantum dots were dissolved in 320 mg mL^−1^ of *n*‐butylamine (BTA) solution.

### PbS‐QDSCs Device Fabrication

The etched ITO‐coated glass was ultrasonically cleaned in a weak detergent solution for 30 min using ultrasound. It was then rinsed three times in deionized water. After, it was ultrasonically cleaned for 15 min using acetone, isopropanol, deionized water, sequentially, and dried. The cleaned ITO‐coated glass substrates were treated by oxygen plasma activation 5 min. Then, the ZnO solution were spin‐coated on ITO substrate at 5000 rpm for 10 s. The dry PbS‐PbX_2_ QDs were dissolved in BTA by 320 mg mL^−1^, the concentration could be changed to account for variations in thickness. After that, the thick quantum dots were spin‐coated onto ZnO films at 1800 rpm for 15 s. The resultant wet PbS‐PbX_2_ QDs films were annealed at 80 °C for 10 min in a glove box. Subsequently, the PEIE solution in isopropanol (0.016% volume concentration) was spin‐coated on the PbS‐PbX_2_ QDs films at 2500 rpm for 40 s. And the films were annealed at 80 °C for 15 min again to produce PEIE treated solar cells. Afterward, two layers of PbS‐EDT were twirled in the air using the stated method. Lastly, two layers of PbS‐EDT were twirled in the air according to the established procedure. PbS solution had a 40 mg mL^−1^ concentration, while EDT solution had a 0.02% concentration. Finally, the 80 nm Au electrode was thermally evaporated on the PbS‐EDT film under a vacuum of 7.5 × 10^−5^ Pa.

## Conflict of Interest

The authors declare no conflict of interest.

## Supporting information

Supporting Information

## Data Availability

The data that support the findings of this study are available from the corresponding author upon reasonable request.
