# Supplementary material for: Interfacial Heterojunction Enables High Efficient PbS Quantum Dot Solar Cells
Source: Adv Sci (Weinh). 2024 May 2;11(26):2402756. doi: 10.1002/advs.202402756 (PMC11234412; doi:10.1002/advs.202402756)
Supplement: Supplementary file 1 — Supporting Information [file ADVS-11-2402756-s001.pdf]

ADVANCED SCIENCE
Open Access

Supporting Information

for *Adv. Sci.*, DOI 10.1002/advs.202402756

Interfacial Heterojunction Enables High Efficient PbS Quantum Dot Solar Cells

Li Zhang, Yong Chen, Shuang Cao, Defei Yuan, Xu Tang, Dengke Wang, Yajun Gao, Junjie Zhang, Yongbiao Zhao, Xichuan Yang, Zhenghong Lu, Quli Fan and Bin Sun**

Interfacial Heterojunction Enables High efficient PbS Quantum Dot Solar Cells

Li Zhang[1,†], Yong Chen[1,†], Shuang Cao[1], Defei Yuan[1], Xu Tang[1], Dengke Wang[4], Yajun Gao[3], Junjie Zhang[1], Yongbiao Zhao[4], Xichuan Yang[2], Zhenghong Lu[4], Quli Fan[1,*], Bin Sun[1,*]

1 State Key Laboratory of Organic Electronics and Information Displays & Institute of Advanced Materials (IAM), School of Material Science and Engineering, Nanjing University of Posts and Telecommunications (NJUPT), 9 Wenyuan Rd., Nanjing 210023, China

2 Institute of Artificial Photosynthesis, State Key Laboratory of Fine Chemicals, DUT−KTH Joint Education and Research Centre on Molecular Devices, Dalian University of Technology (DUT), 2 Linggong Rd., Dalian 116024, China

3 LONGi Central R&D Institute, LONGi Green Energy Technology Co., Xi'an, China.

4 Department of Physics, Center for Optoelectronics Engineering Research, Yunnan University, Kunming 650091, China

†These authors contributed equally to this work.

(*Email: iamqlfan@njupt.edu.cn; iambsun@njupt.edu.cn)

1. Materials

Lead (II) oxide (Sigma-aldrich, ≥99.9% trace metals basis), Oleic acid (Sigma-aldrich, 90% technical grade), 1-Octadecene (Aladdin, >90.0% Gas chromatography), and Bis(trimethylsilyl)sulfur $(TMS)_2S$ (TCI, 97.0% Gas chromatography), Ethyl acetate (Innochem, 99.8% water ≤ 50 ppm), Ethanol (Aladdin, 95% Analytical reagent) were employed as received for PbS QD synthesis and purification. Lead (II) iodide (Advanced Election Co., Ltd (China), 99.999% Super dry large crystals), Lead (II) bromide ammonium (Aladdin, 99.999% metals basis) acetate (Innochem, ≥ 99% Guaranteed reagent), and N, N-dimethylformamide (DMF) (Innochem, 99.8% SafeDry), 1,2-Ethanedithiol (TCI 99%), Acetonitrile (Innochem 99.9% Water ≤50 ppm) were used in the ligand exchange. Polyethylenimine (PEIE) (Sigma-aldrich 80% ethoxylated solution), Isopropyl alcohol (Innochem, ≥ 99.9% Gas chromatography). All the materials were used as received unless specified.

2. Preparation of ZnO nanoparticle

A 250 mL three-neck flask was first placed on the oil bath, to which 2.95 g of zinc acetate dihydrate and 125 mL of methanol were added. The solution was then

heated to 61 °C and stirred for a while. After that, 65 mL of a 0.0227 g/mL potassium hydroxide solution containing methanol as the solvent was added. This procedure took 10 to 15 min to complete. The reaction was full for 2.5 h following the addition of the potassium hydroxide solution. After 3 h of standing, discard the supernatant and mix the ZnO precipitate thoroughly with 50 mL methanol. The first wash was washed with 50 mL methanol, shook 90 s and centrifuged at 2500 rpm/2 min. The second wash was also shaken 90 s and centrifuged at 6000 rpm/3 min. Dissolve the solid with 4 mL trichloromethane and then add 16 mL methanol to form 20 mL solution.

3. Preparation of PbS QDs

The primary technique for creating quantum dots is hot injection, which involves adding 2.25 g of PbO and 8 mL of OA to a 100 mL flask after 20 mL of ODE has been added. Before heating the solution to 85 °C, the entire flask was vacuumed for 1 h, then for 5 min, high purity nitrogen was added, and finally for 5 min of vacuuming. After the allotted time has elapsed, the temperature is increased to 122°C and the reaction is allowed to continue for an additional 40 min at 85°C. The solution should typically be transparent or have a yellowish hue if the reaction is normal. After dissolving 700 μL of $(TMS)_2S$ in 5 mL of dehydrated ODE, the mixture was promptly injected into a flask containing nitrogen of the highest purity. For the synthesis of quantum dots, the main method is hot injection, which first adds 20 mL ODE to 100 mL flask, then adds 8ml OA and 2.25 g PbO. The whole flask was vacuumed for 1 h, followed by high purity nitrogen for 5 min, and then vacuumed for another 5 min before heating the solution to 85 °C. Reaction at 85 °C for 40 min, after the time is up, the temperature is raised to 122 °C, and the reaction continues for 40 min. Normally, if the reaction is normal, the solution should be transparent or yellowish in color. 700 μL of $(TMS)_2S$ was then dissolved in 5 mL of dehydrated ODE, and the solution was quickly injected into a flask with high purity nitrogen. The solution changed from transparent to black, and the reaction lasted for 1 min. After the reaction time expires, the flask is quickly lifted and 20 mL of n-hexane is injected for quenching reaction. The reaction solution is poured into the centrifuge tube and left for one night at 1~3 °C. The next day, the unreacted PbO and other impurities were removed by centrifugation at 6000 rpm/5min. The quantum dots were washed three times with a mixture of 1:2 ethyl acetate and ethanol, and vacuum for 5 h to obtain the required high-purity quantum dots. All other operations stay the same, except for lowering the injection temperature to 100 °C to produce the smaller quantum dots required for the EDT layer.

4. PbS-PbX_2 Ligand Exchange

PbI_2 (0.1 M), $PbBr_2$ (0.04 M), and ammonium acetate (0.04 M) are dissolved in 20 mL DMF for a standard ligand exchange. The 7 mg/mL PbS (20 mL) solution was mixed with the DMF solution. Shake vigorously for 2 min. During this period, the ligand exchange event causes the upper liquid to turn colorless and the lower liquid to turn black. Rinse five times with 20mL of n-hexane. After adding toluene at a volume ratio of 1:3, the precipitate was finally achieved, and the PbS quantum dots were dried in a vacuum for 20 min. After that, the dried quantum dots were dissolved in 320 mg/mL of n-butylamine (BTA) solution.

5. PbS-QDSCs Device Fabrication

The etched ITO-coated glass was ultrasonically cleaned in a weak detergent solution for 30 minutes using ultrasound. It was then rinsed three times in deionized water. After, it was ultrasonically cleaned for 15 min using acetone, isopropanol, deionized water, sequentially, and dried. The cleaned ITO-coated glass substrates were treated by oxygen plasma activation 5 min. Then, the ZnO solution were spin-coated on ITO substrate at 5000 rpm for 10 s. The dry PbS-PbX_2 QDs were dissolved in BTA by 320 mg/mL, the concentration could be changed to account for variations in thickness. After that, the thick quantum dots were spin-coated onto ZnO films at 1800 rpm for 15 s. The resultant wet PbS-PbX_2 QDs films were annealed at 80 °C for 10 min in a glove box. Subsequently, the PEIE solution in isopropanol (0.016% volume concentration) was spin-coated on the PbS-PbX_2 QDs films at 2500 rpm for 40 s. And the films were annealed at 80 °C for 15 min again to produce PEIE treated solar cells. Afterward, two layers of PbS-EDT are twirled in the air using the stated method. Lastly, two layers of PbS-EDT are twirled in the air according to the established procedure. PbS solution has a 40 mg/mL concentration, while EDT solution has a 0.02% concentration. Finally, the 80 nm Au electrode was thermally evaporated on the PbS-EDT film under a vacuum of 7.5×10^{-5} Pa.

6. PbS-QDSCs Characterization

The *J-V* curves were measured by Keithley 2400 source meter. Illumination was provided by an Oriel Sol3A solar simulator (SS-F53A, Enli Technology Co. Ltd, Taiwany) with AM 1.5G spectrum and light intensity of 100 mW/cm^2, which was determined by a calibrated crystalline Si-cell. Simultaneously, a mask is added to the test. Each mask has eight holes with an area of 0.048 cm^2, which is used to evaluate the solar cell's current density more precisely. The devices were measured in reverse scan from 0.7 V to -0.7 V with 0.02 V intervals and 10 ms delay time.

By applying various filters with known optical densities to the solar cell being tested, the intensity-dependent *J*-*V* curve may be derived (ThorLabs Absorbing ND Filter Kit). The QE system (EnliTech) is used to measure EQE in ambient air. Monochromatic light is focused on device pixels at a chopper frequency of 20 Hz. EQE spectra were acquired at zero electrical bias, whereas IQE spectra were calculated from EQE spectra taken at a negative bias of -1 V using the following formula: IQE = EQE (0 V)/EQE (-1 V).

7. Absorbance and Luminescence

PbS quantum dots were prepared on glass by spin coating method, and a PEIE layer was added on the PbS quantum dots layer. The absorption spectra were measured by PerkinElmer Lamda650. Edinburgh FLS920 was used to test the PL of thin films and the PL of solar cells. Xe lamp was used as the excitation source to excite the thin films, and NIR-PMT (300-1700 nm) was used as the detection unit to collect PL signals.

8. X-ray diffraction

PbS films drop-cast were analyzed with a Bruker D8 Advance A25 Discover diffractometer equipped with a 1.6 kW Cu x-ray filament (λ = 1.5 Å) and a 0.6 mm slit. Scanning was performed with a 1D detector in $\theta/2\theta$ mode.

9. X-ray spectroscopy

XPS measurements were performed in an ultra-high vacuum chamber (ESCALAB 250Xi, supplied by Thermo Scientific, base pressure: 2×10^{-10} mbar) using an XR6 monochromatic Al Kα source (hν = 1486.6 eV) with a flux energy of 20 eV. XPS depth profiling was carried out using a MAGCIS dual mode Ion Source to produce a large argon cluster (Ar2000) argon cluster ion beam with an energy of 4 keV for XPS depth profiling. XPS spectra were evaluated using Thermo Scientific's advantage software. First, a suitable background was added to each spectrum, and then the peaks were fitted using a Gauss-Lorentz function. The fit of the double peaks was suppressed in terms of spin-orbit splitting, peak area ratio and half-height width. Finally, the calculated peak areas and their corresponding atomic sensitivity factors were used to calculate the proportions of the different elements, with the Control group as well as the With Peie group using element S as the reference element.

10. UPS spectroscopy

UPS measurements were performed using a He gas discharge lamp emitting He I radiation (hv = 21.22 eV) with a pass energy of 10 eV and a bias of -9 V to ensure secondary electron onset detection. Cluster etching was performed using large Ar clusters generated by the GCIS lon Source (Kratos Analytical Inc.) with an energy of

10 keV.

Take the calculation of PbS-PbX_2 films energy level as an example. The Fermi energy levels of the films are all determined by the secondary electron cutoffs, where the Fermi energy level of film calculated by equation E_F = 21.2 eV-E_{cutoff} (E_{cutoff} is secondary electron cutoff energy), the E_F. is obtained as 4.61 eV. Addition, according to the valence band formula $E_{onset} = E_F$-E_{VBM}, the E_{VBM} is obtained as -5.44 eV. (E_{onset} is secondary electron starting edge energy, E_{VBM} is energy of the valence band maximum). At the same time according to the formula E_g=1240/λ_g (eV), where E_g is the quantum dot optical film band gap, λ_g is the absorption peak of the quantum dot film, the E_g is obtained as 1.29 eV. According to the formula $E_g = E_{CBM} - E_{VBM}$, (E_{CBM} is the conduction band bottom of the film) E_{CBM} is obtained as -4.15 eV. Similarly, the E_F of the PbS-PbX_2+PEIE film is calculated to be 4.47 eV, the valence band top energy level to be -5.37 eV, and the conduction band bottom energy level to be -4.09 eV.

11. AFM Measurements

The AFM images were obtained by photographing the film surface with Bruker's Dimension Icon model AFM.

12. SEM Measurements

The cross-section image of the device was investigated using a Hitach S-4800 SEM.

13. EIS Testing

Ac impedance spectroscopy was measured using an impedance analyzer (Metrohm AUTOLAB PGSTAT302N) under open circuit conditions. Frequency ranges from 0.1 kHz to 1 MHz Using EC-lab software to fit the data, the equivalent circuit diagram is determined.

14. TPV Testing

TPV measurements were performed on the Paios, Fluxim AG. TPV curves were measured at 100% offset Intensity, with a pulse length of 15 s, and a follow-up time of 5 s.

15. TA Testing

The experimental setup for ultrafast spectroscopic and kinetic measurements. The 800 nm excitation pulses (120 fs, 1000 Hz) were generated by an amplified Ti: sapphire laser system (Spectra Physics). The transient absorption setup (Helios, Ultrafast Systems) is characterized by a temporal resolution of ca. 170 fs and spectral resolution of 5 nm. The kinetic curves were fitted by single wavelength.[1-2] Excitation wavelength of sample is 500 nm, power 180 μW. All the measurements were

performed at room temperature under aerated conditions. It's worth emphasizing that the sample is fixed on a movable sample rack (like a detector) for moving test to ensure that the laser is not repeated at the same point to avoid damage to the sample. The decay was fitted using the following Equation (1):

$$I(t) = A \cdot exp\left(-\frac{t}{\tau_1}\right) + B \cdot exp\left(-\frac{t}{\tau_2}\right) + C \cdot exp\left(-\frac{t}{\tau_3}\right) + D \qquad \text{(Eq. 1)}$$

where *A*, *B*, and *C*, *D* are constants, *t* is decay time, τ_1, τ_2 and τ_3 are fitted lifetimes.

16. J_{sc} and V_{oc} versus light intensity curves calculation

The J_{sc} is expected to demonstrate a power-law dependence on light intensity I ($J_{sc} \approx I^{\alpha}$). And for the light intensity dependence on V_{oc}, if the slope is greater than 1kT/q (where k is the Boltzmann constant, T is the temperature, and q is the elementary charge), it indicates the interfacial trap-assisted Shockley−Read−Hall (SRH) recombination. These values are close to 1, suggesting that the extraction of charge carriers is primarily limited by bimolecular recombination. The V_{oc} ought to possess a liner correlation with the logarithm of light intensity according to Equation (2).

$$(\mathrm{d}\ V_{oc})/\mathrm{dlgI} = 2.303\ (nk_B\ \mathrm{T})/\mathrm{q} \qquad (2)$$

where k_B is the Boltzmann constant, *n* is the ideality factor, T is the thermodynamic temperature, and q is the elementary charge.

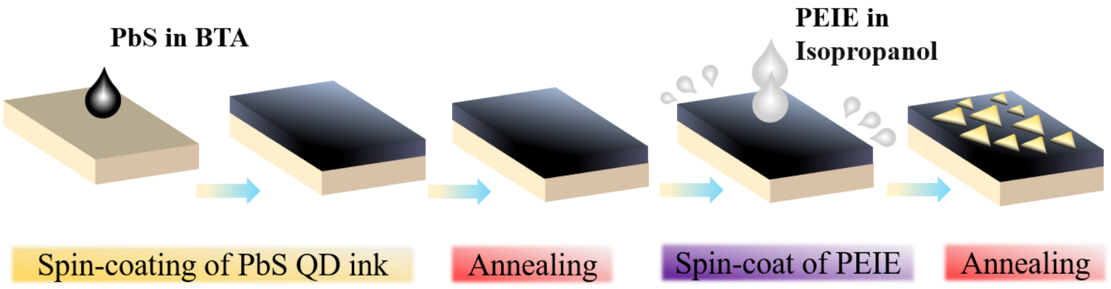

Figure S1 The process of CQDs absorption layer and PEIE preparation.

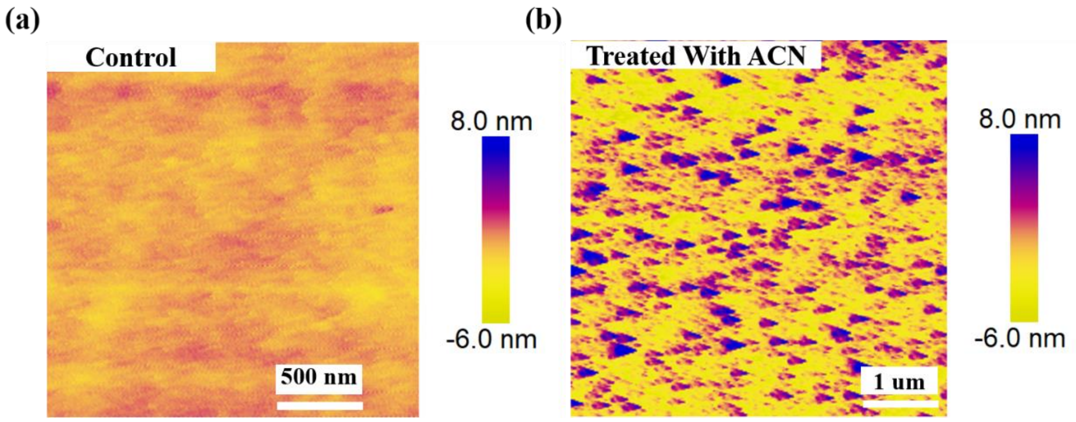

Figure S2 (a) AFM images of control ($PbS-PbX_2$) films on ITO substrates. (b) AFM images of with PEIE ($PbS-PbX_2$+PEIE) films cleaned by acetonitrile.

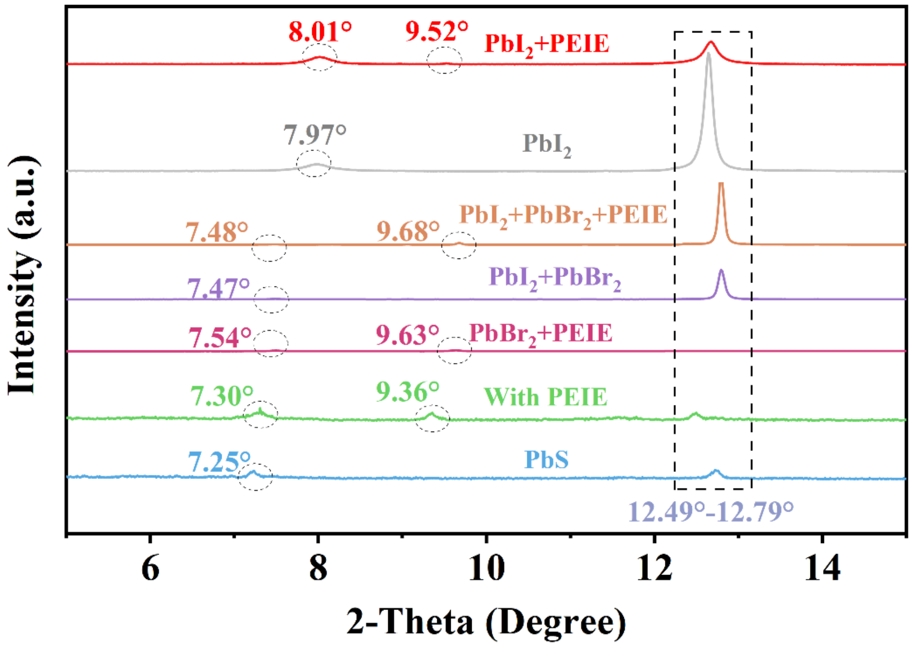

Figure S3. XRD spectra of PbI_2+PEIE, PbI_2, PbI_2+$PbBr_2$+PEIE, PbI_2+$PbBr_2$, $PbBr_2$+PEIE, PbI_2+$PbBr_2$, PbI_2+$PbBr_2$+PEIE, control ($PbS-PbX_2$) and with PEIE ($PbS-PbX_2$+PEIE) films at a small Angle in 5-15°.

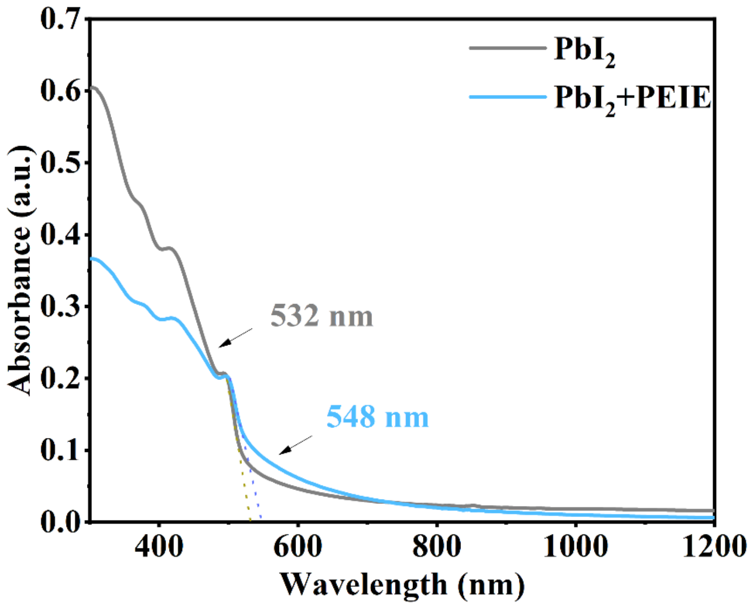

Figure S4. UV-Vis absorption spectra of (a) PbI_2 and PbI_2+PEIE films.

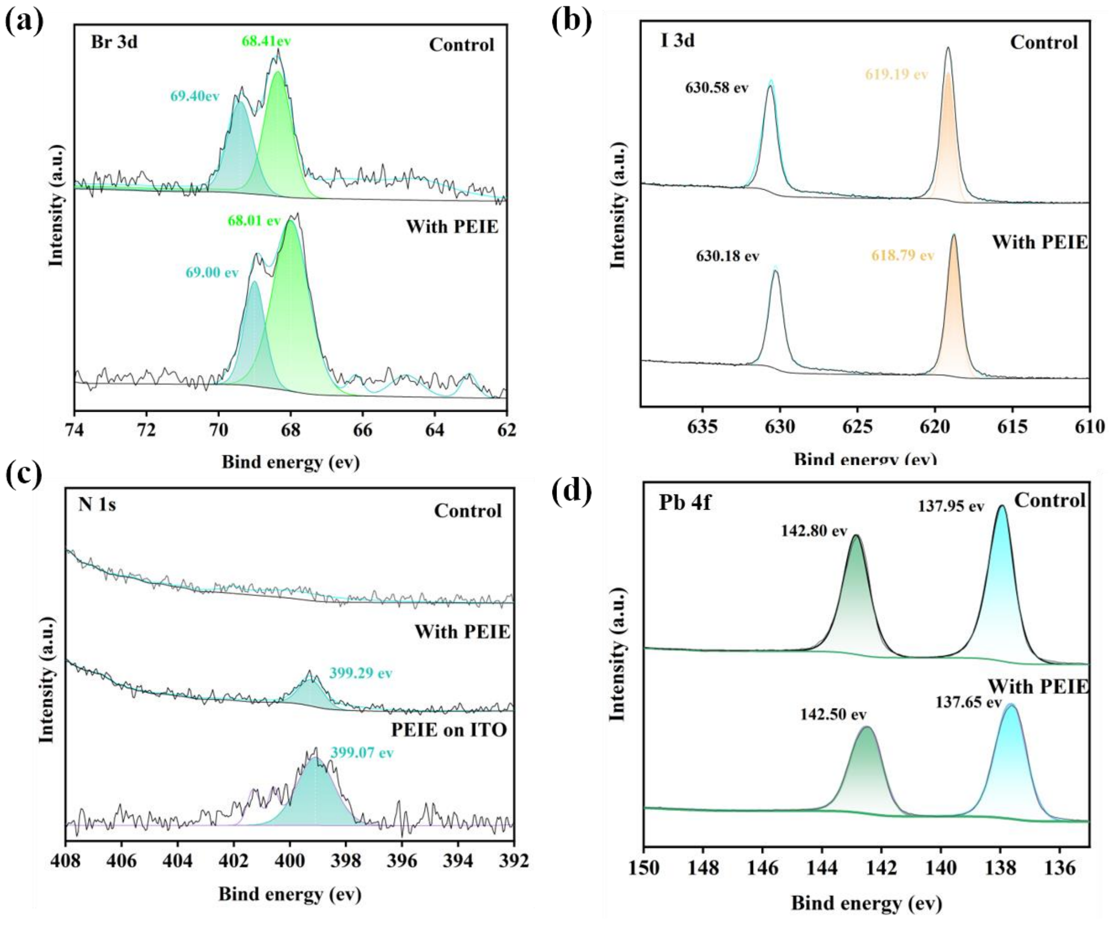

Figure S5. XPS spectra of (a) Br 3d, (b) I 3d and (c) N 1s (d) Pb 4f for control and with PEIE films.

Compared with the control film, PEIE on ITO and with PEIE sample shows a N 1s peak and slight shift, while bare PbS film displays no signal (Figure S5). And the N

1s peak of with PEIE film to higher binding energy direction. The Br 3d, I 3d and Pb 2p peaks of with PEIE film shift to lower binding energy direction. other elements did not shift significantly with the PbS-PbX_2 and PbS-PbX_2+PEIE film. It implies that the modulation of PEIE and interaction with control film via some kind chemical action.

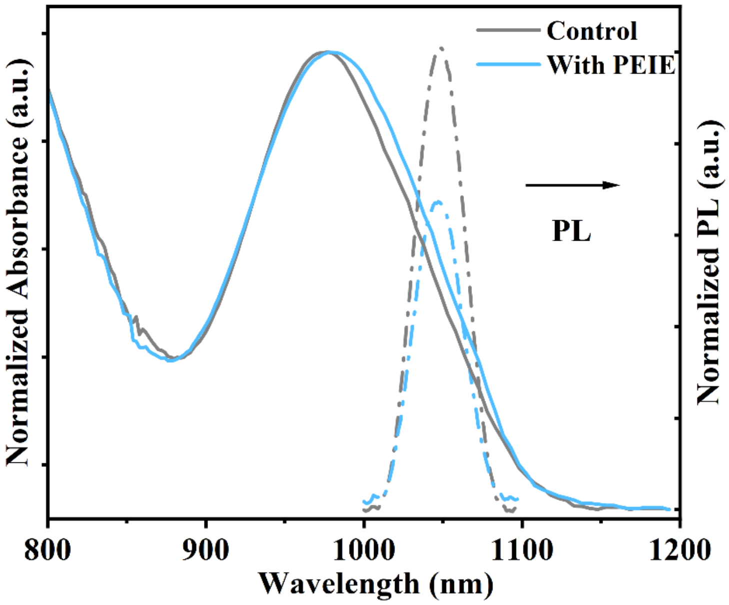

Figure S6. Normalized UV-vis absorption and photoluminescence of control (PbS-PbX_2) and with PEIE (PbS-PbX_2+PEIE) films.

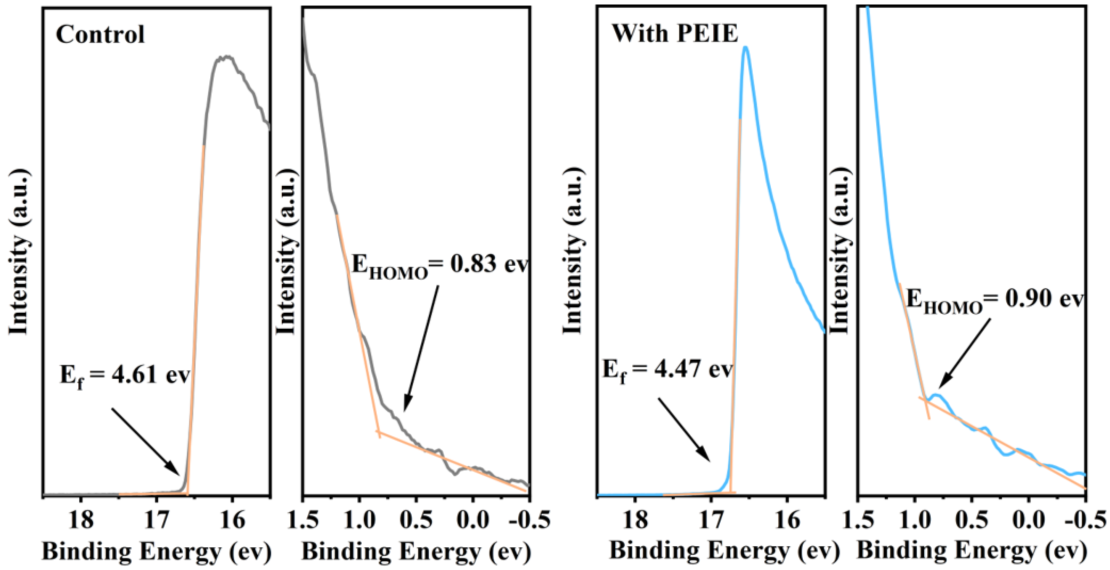

Figure S7. UPS spectra for control (PbS-PbX_2) and with PEIE (PbS-PbX_2+PEIE) films, respectively.

The fermi energy (E_F) levels of control (PbS-PbX_2) and with PEIE (PbS-PbX_2+PEIE) films are calculated to be 4.61 and 4.47 eV, respectively, and E_F of PbS-PbX_2+PEIE is further away from to the valence band maximum (VBM) than PbS-PbX_2 (the corresponding energy gap between E_F and VBM are 0.83 and 0.90 eV,

respectively). As shown in Figure S7, the VBM levels of PbS-PbX_2 and PbS-PbX_2+PEIE are -5.44 and -5.37 eV, respectively. It is noted that the reduction of the energy level offset (ΔE) leads to lower charge accumulation at the interface, which can effectively increase the charge extraction/transport efficiency and suppress non-radiative charge recombination.

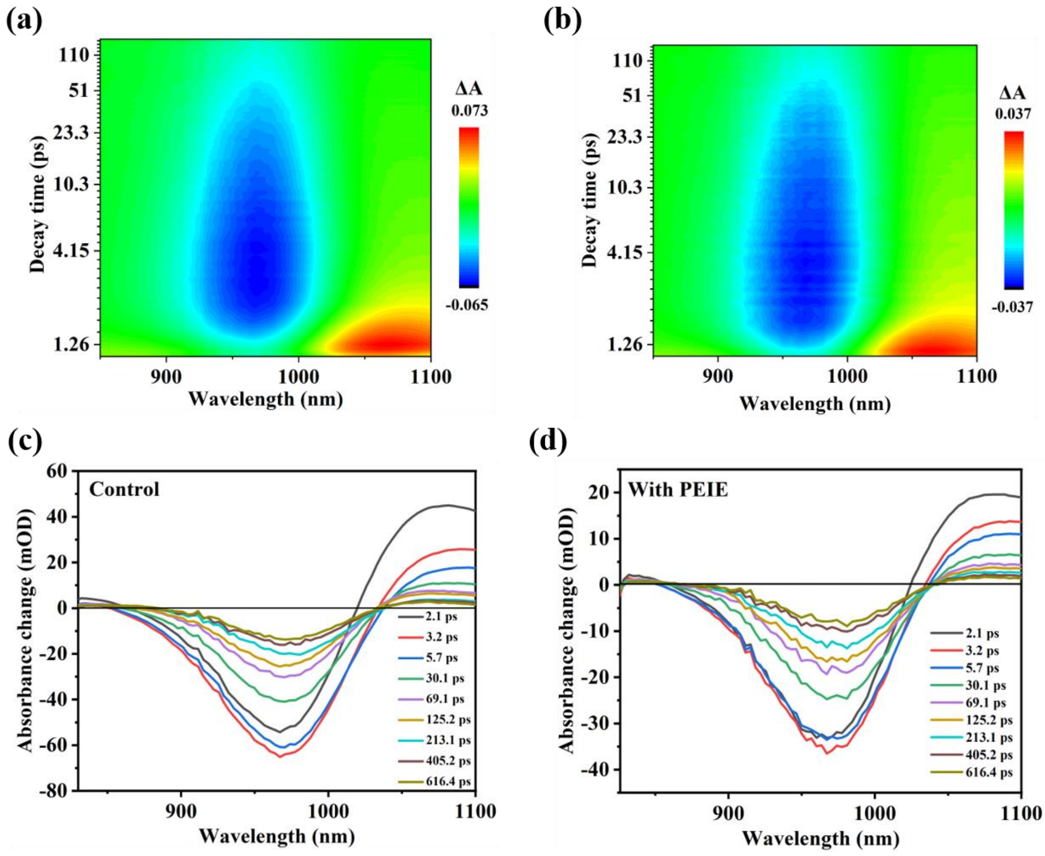

Figure S8. (a-b) The contour plots of the ps-TA spectra of control (PbS-PbX_2) and with PEIE (PbS-PbX_2+PEIE) film samples as functions of wavelength and delay time. (excitation wavelength 500 nm, excitation intensity 180 μw.). (c-d) The fs-TA spectral profiles from 2.1 ps to 616.4 ps of (c) control, (d) with PEIE samples.

Table S1 Summary of the charge lifetime parameters from fitting curves of the TA decay measurements

λ & Laser power	Sample	A_1	τ_1(ps)	A_2	τ_2(ps)	A_3	τ_3(ps)
NIR-ex500-180uw	control	0.555	0.7270	0.260	20.28	0.184	352.53
NIR-ex500-180uw	with PEIE	0.526	0.4579	0.278	36.65	0.195	559.64

Table S2. Photovoltaic performance of PbS CQD solar cells based on CTL (control PbS-QDSCs) and HTJ (heterojunction PbS-PEIE QDSCs) devices under AM 1.5G

Simulated Sunlight [a]

Devices		V_{oc} (V)	J_{sc} (mA·cm^{-2})	FF (%)	PCE (%)
CTL	Champion	0.64	27.5	69.5	12.2
HTJ	Champion	0.65	29.6	69.7	13.4

[a] The working area of the cells is 0.048 cm^2.

Table S3. EIS parameters of PbS CQD solar cells based on CTL (control PbS-PbX_2 CQD solar cell) and HTJ (heterojunction PbS-PbX_2+PEIE CQD solar cell) devices.

Devices	R_{tr} (Ω)	R_s (Ω)	C_μ (nF)
CTL	28.65	28.04	3.89
HTJ	1493	1268	3.89

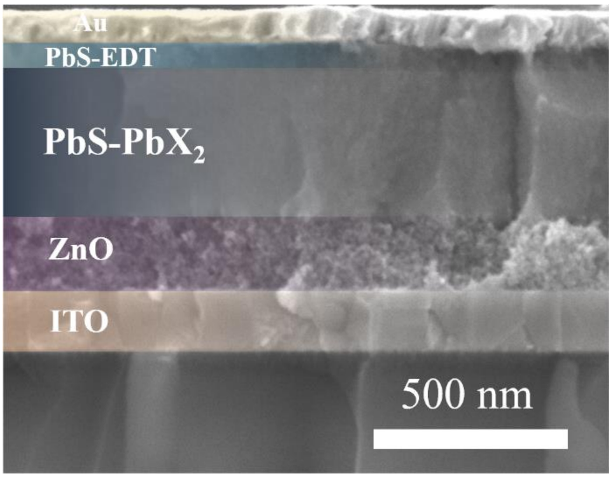

Figure S9. Cross-sectional SEM image of the CTL devices. (~50 nm of EDT-exchanged PbS CQD film)

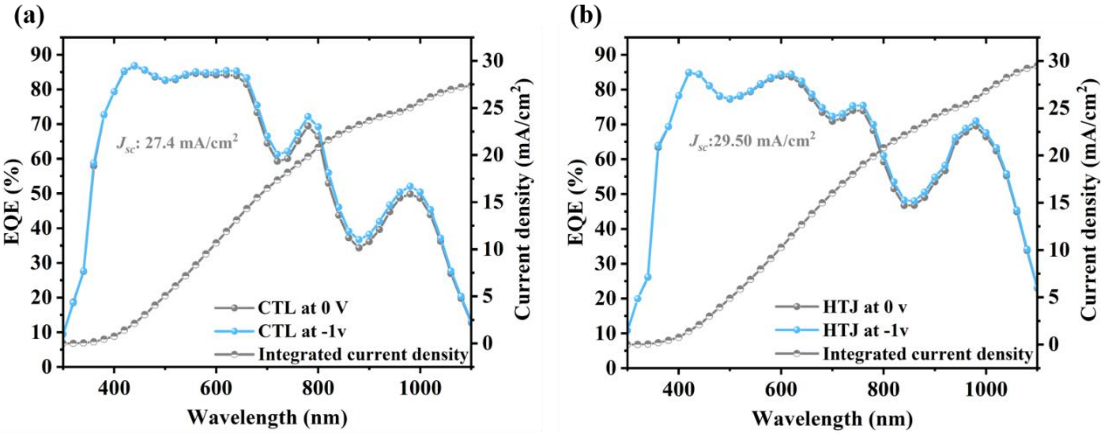

Figure S10. External quantum efficiency (EQE) curves of CTL and HTJ devices under bias voltage 0 V and -1V. (Due to equipment limitations, only ~1100 nm was measured)

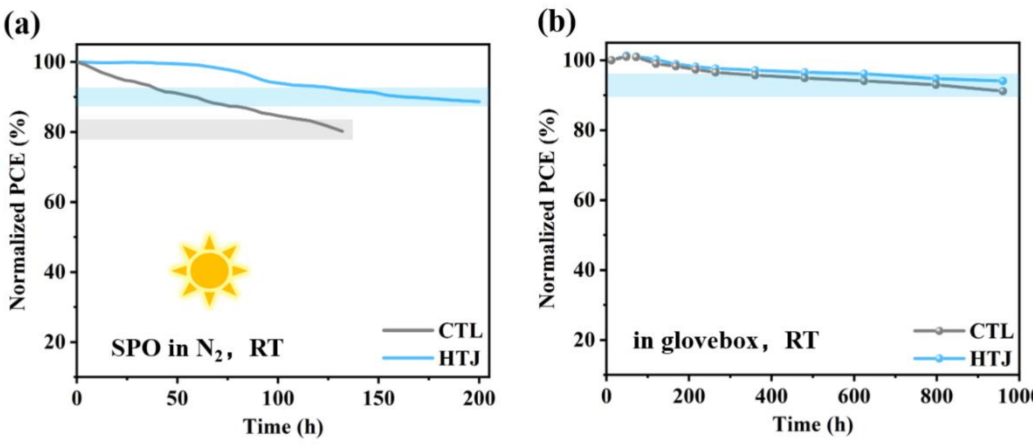

Figure S11. (a) The stabilized power output stability of unencapsulated device at a maximum power point under a continuous AM 1.5 G illumination in a N_2 condition. (b) The stability of natural conditions in glovebox for 1000 h.

Table S4. The key parameters of different solar cells in various interface engineering.

NO.	V_{oc} (V)	J_{sc} (mA cm^{-2})	FF (%)	PCE (%)	Materials/ Method	Ref.
1 QDSCs	0.65	30.00	71.0	13.80	FAI or FABr	[3]
2 QDSCs	0.65	27.93	66.2	12.02	PTB7-Th: PC71BM	[4]
3 QDSCs	0.60	28.37	65.8	11.20	PBDB-T(F)	[5]
4 QDSCs	0.61	31.10	57.0	10.80	H-plasma	[6]
5 QDSCs	0.67	32.44	67.0	13.74	EDT/PBDTTT-E-T	[7]
6 QDSCs	0.68	28.64	69.5	13.54	TPAOMe-C8	[8]
7 QDSCs	0.63	27.80	65.3	11.60	Cl/ZnO	[9]
8 QDSCs	0.66	29.60	67.0	13.10	Polymer-SM-bridge	[10]
9 QDSCs	0.65	28.37	65.6	12.14	$CsPbI_3$	[11]
10 QDSCs	0.57	25.30	65.0	9.30	MgZnO	[12]
11 QDSCs	0.60	29.50	43.0	7.50	CdSe QD	[13]
12 QDSCs	0.66	31.5	74.0	15.4	PMMA: PCBM	[14]
13 QDSCs	0.60	32.4	55.0	10.7	BHJ	[15]
14 QDSCs	0.60	30.2	58.0	10.5	NH_4SCN	[16]
15 QDSCs	0.60	30.3	52.0	9.6	3-methyl mercapto	[17]
16 QDSCs	0.59	28.9	66.0	11.3	CSMAFA	[18]

17 QDSCs	0.68	24.11	67.0	10.99	PDMII	[19]
18 QDSCs	0.63	29.61	66.7	12.42	Ag	[20]
19 QDSCs	0.62	27.98	65.0	11.29	PEAI	[21]
20 Ternary OSCs	0.89	19.30	75.9	13.40	PBT1-C: ICBA: ITIC-2Cl	[22]
21 PSCs	1.20	22.72	77.5	21.06	EABr	[23]
22 PSCs	1.14	24.60	83.8	23.70	4Tel	[24]
23 PSCs	1.30	13.13	70.4	12.02	P3HT	[25]
24 PSCs	1.25	14.89	75.3	14.00	CsAc	[26]
25 QDSCs	0.65	29.6	69.7	13.4	PEIE	This work

References:

1. X. Li, Ch. Gong, G. G. Gurzadyan, M. F. Gelin, J. Liu, L.Sun, *J. Phys. Chem. C*, **2018**, *122*, 50.

2. S. Y. Chen, Y. H. Pan, K. Chen, P. F. Chen, Q. M. Shen, P. F. Sun, W. B. Hu, Q. L. Fan, *Angew. Chem.Int. Ed.* **2023**, 62, e2022153.

3. B. Sun, A. Johnston, C. Xu, M. Wei, Z. Huang, Z. Jiang, H. Zhou, Y. Gao, Y. Dong, O. Ouellette, X. Zheng, J. Liu, M.-J. Choi, Y. Gao, S.-W. Baek, F. Laquai, O. M. Bakr, D. Ban, O. Voznyy, F. P. García de Arquer, E. H. Sargent, *Joule* **2020**, 4 (7), 1542.

4. Y. Zhang, Y. Kan, K. Gao, M. Gu, Y. Shi, X. Zhang, Y. Xue, X. Zhang, Z. Liu, Y. Zhang, J. Yuan, W. Ma, A. K. Y. Jen, *ACS Energy Lett.* **2020**, 5 (7), 2335.

5. Y. Xue, F. Yang, J. Yuan, Y. Zhang, M. Gu, Y. Xu, X. Ling, Y. Wang, F. Li, T. Zhai, J. Li, C. Cui, Y. Chen, W. Ma, *ACS Energy Lett.* **2019**, 4 (12), 2850.

6. H. Tavakoli Dastjerdi, D. Prochowicz, P. Yadav, M. M. Tavakoli, *Adv. Mater. Interfaces* **2019**, 7 (1), 1901551.

7. B. Kim, S. W. Baek, C. Kim, J. Kim, J. Y. Lee, *Adv. Energy Mater.* **2021**, 12 (2), 2102689.

8. S. Fang, J. Huang, R. Tao, Q. Wei, X. Ding, S. Yajima, Z. Chen, W. Zhu, C. Liu, Y. Li, N. Yin, L. Song, Y. Liu, G. Shi, H. Wu, Y. Gao, X. Wen, Q. Chen, Q. Shen, Y. Li, Z. Liu, Y. Li, W. Ma, *Adv. Mater.* **2023**, 35 (21), 2212184.

9. J. Choi, Y. Kim, J. W. Jo, J. Kim, B. Sun, G. Walters, F. P. García de Arquer, R. Quintero-Bermudez, Y. Li, C. S. Tan, L. N. Quan, A. P. T. Kam, S. Hoogland, Z. Lu, O. Voznyy, E. H. Sargent, *Adv. Mater.* **2017**, 29 (33), 1702350.

10. S.-W. Baek, S. Jun, B. Kim, A. H. Proppe, O. Ouellette, O. Voznyy, C. Kim, J. Kim, G. Walters, J. H. Song, S. Jeong, H. R. Byun, M. S. Jeong, S. Hoogland, F. P. García de Arquer, S. O. Kelley, J.-Y. Lee, E. H. Sargent, *Nat. Energy* **2019**, 4 (11), 969.

11. X. Meng, Y. Chen, F. Yang, J. Zhang, G. Shi, Y. Zhang, H. Tang, W. Chen, Y. Liu, L. Yuan, S. Li, K. Wang, Q. Chen, Z. Liu, W. Ma, *Nano Research* **2022**, 15 (7), 6121.

12. X. Zhang, E. M. J. Johansson, *J. Mater. Chem. A* **2017**, 5 (1), 303.

13. T. Zhao, E. D. Goodwin, J. Guo, H. Wang, B. T. Diroll, C. B. Murray, C. R. Kagan, *Acs Nano* **2016**, 10 (10), 9267.

14. C. Ding, D. Wang, D. Liu, H. Li, Y. Li, S. Hayase, T. Sogabe, T. Masuda, Y. Zhou, Y. Yao, Z. Zou, R. Wang, Q. Shen, *Adv. Energy Mater.* **2022**, 12 (35), 2201676.

15. N. V. Dambhare, A. Sharma, C. Mahajan, A. K. Rath, *Energy Technol.* **2022**, 10 (9), 2200455.

16. A. Sharma, N. V. Dambhare, J. Bera, S. Sahu, A. K. Rath, *Acs Appl. Nano Mater.* **2021**, 4 (4), 4016.

17. A. Sharma, C. Mahajan, A. K. Rath, *Acs Appl. Energy Mater.* **2020**, 3 (9), 8903.

18. M. Albaladejo-Siguan, D. Becker-Koch, A. D. Taylor, Q. Sun, V. Lami, P. G. Oppenheimer, F. Paulus, Y. Vaynzof, *Acs Nano* **2020**, 14 (1), 384.

19. R. Azmi, S. Sinaga, H. Aqoma, G. Seo, T. K. Ahn, M. Park, S.-Y. Ju, J.-W. Lee, T.-W. Kim, S.-H. Oh, S.-Y. Jang, *Nano Energy* **2017**, 39, 86.

20. J. Li, X. Zhang, Z. Liu, H. Wu, A. Wang, Z. Luo, J. Wang, W. Dong, C. Wang, S. Wen, Q. Dong, W. W. Yu, W. Zheng, *Small* **2024**, 2311461.

21. X. Yang, J. Yang, M. I. Ullah, Y. Xia, G. Liang, S. Wang, J. Zhang, H.-Y. Hsu, H. Song, J. Tang, *Acs Appl. Mater. Interfaces* **2020**, 12 (37), 42217.

22. Y. Xie, F. Yang, Y. Li, M. A. Uddin, P. Bi, B. Fan, Y. Cai, X. Hao, H. Y. Woo, W. Li, F. Liu, Y. Sun, *Adv. Mater.* **2018**, 30 (38), 1803045.

23. J. Ren, T. Liu, B. He, G. Wu, H. Gu, B. Wang, J. Li, Y. Mao, S. Chen, G. Xing, *Small* **2022**, 18 (47), 2203536.

24. J. Sun, K. Ma, Z. Y. Lin, Y. Tang, D. Varadharajan, A. X. Chen, H. R. Atapattu, Y. H. Lee, K. Chen, B. W. Boudouris, K. R. Graham, D. J. Lipomi, J. Mei, B. M. Savoie, L. Dou, *Adv. Mater.* **2023**, 35 (26), 2300647

24. Q. Zeng, X. Zhang, X. Feng, S. Lu, Z. Chen, X. Yong, S. A. T. Redfern, H. Wei, H. Wang, H. Shen, W. Zhang, W. Zheng, H. Zhang, J. S. Tse, B. Yang, *Adv. Mater.* **2018**, 30 (9), 1705393.

25. X. Ling, S. Zhou, J. Yuan, J. Shi, Y. Qian, B. W. Larson, Q. Zhao, C. Qin, F. Li, G. Shi, C. Stewart, J. Hu, X. Zhang, J. M. Luther, S. Duhm, W. Ma, *Adv. Energy Mater.* **2019**, 9 (28), 1900721.
